# Can levosimendan reduce ECMO weaning failure in cardiogenic shock?: a cohort study with propensity score analysis

**DOI:** 10.1186/s13054-020-03122-y

**Published:** 2020-07-16

**Authors:** Enrique Guilherme, Matthias Jacquet-Lagrèze, Matteo Pozzi, Felix Achana, Xavier Armoiry, Jean-Luc Fellahi

**Affiliations:** 1 Hospices Civils de Lyon, Hôpital Louis Pradel, Service d’Anesthésie-Réanimation, Lyon, France; 2 INSERM U1060, Laboratoire CarMeN, IHU OPeRa, Lyon, France; 3 Hospices Civils de Lyon, Hôpital Louis Pradel, Service de Chirurgie Cardiaque, Lyon, France; 4 Nuffield Department of Primary care, Oxford University, Oxford, UK; 5 Lyon School of Pharmacy (ISPB), Public Health department/UMR CNRS 5510 MATEIS, I2B Team, Lyon, France; 6 Division of Health Sciences, Warwick Medical School, Warwick university, Coventry, UK

**Keywords:** Levosimendan, VA-ECMO, Cardiogenic shock, ECMO weaning failure, Circulatory failure

## Abstract

**Background:**

Veno-arterial extracorporeal membrane oxygenation (VA-ECMO) has been increasingly used over the last decade in patients with refractory cardiogenic shock. ECMO weaning can, however, be challenging and lead to circulatory failure and death. Recent data suggest a potential benefit of levosimendan for ECMO weaning. We sought to further investigate whether the use of levosimendan could decrease the rate of ECMO weaning failure in adult patients with refractory cardiogenic shock.

**Methods:**

We performed an observational single-center cohort study. All patients undergoing VA-ECMO from January 2012 to December 2018 were eligible and divided into two groups: group levosimendan and group control (without levosimendan). The primary endpoint was VA-ECMO weaning failure defined as death during VA-ECMO treatment or within 24 h after VA-ECMO removal. Secondary outcomes were mortality at day 28 and at 6 months. The two groups were compared after propensity score matching. *P* < 0.05 was considered statistically significant.

**Results:**

Two hundred patients were analyzed (levosimendan group: *n* = 53 and control group: *n* = 147). No significant difference was found between groups on baseline characteristics except for ECMO duration, which was longer in the levosimendan group (10.6 ± 4.8 vs. 6.5 ± 4.7 days, *p* < 0.001). Levosimendan administration started 6.6 ± 5.4 days on average following ECMO implantation. After matching of 48 levosimendan patients to 78 control patients, the duration of ECMO was similar in both groups. The rate of weaning failure was 29.1% and 35.4% in levosimendan and control groups, respectively (OR: 0.69, 95%CI: 0.25–1.88). No significant difference was found between groups for all secondary outcomes.

**Conclusion:**

Levosimendan did not improve the rate of successful VA-ECMO weaning in patients with refractory cardiogenic shock.

**Trial registration:**

ClinicalTrials.gov, NCT04323709.

## Introduction

Veno-arterial extracorporeal membrane oxygenation (VA-ECMO) is a temporary mechanical circulatory support that has been increasingly used over the last decade to restore and maintain adequate end-organ perfusion and improve outcomes in patients with refractory cardiogenic shock [1, 2]. Nevertheless, the weaning of VA-ECMO should be daily questioned, as several studies reported severe complications like cannula-related infections [3], bleeding [4], and thromboembolic events [5] associated with prolonged VA-ECMO durations. Dobutamine is currently used to improve myocardial contractility during VA-ECMO, aiming to enhance left ventricular ejection and aortic valve opening and also to shorten ECMO duration. Numerous data suggest however an increased risk of mortality related to myocardial ischemia and arrhythmias [6, 7]. Levosimendan is a calcium-sensitizing inotropic agent with systemic, coronary, and pulmonary vasodilatory properties and also specific cardioprotective effects with respect to myocardial oxygen balance [8–10]. It has been approved for the treatment of acute decompensated heart failure, but its efficacy in cardiogenic shock remains questionable [11]. The use of levosimendan in patients undergoing VA-ECMO might be of interest both to reduce the duration of mechanical support and to minimize severe complications. A potential benefit in terms of VA-ECMO weaning success and increased survival has been recently suggested in low cardiac output syndrome following cardiac surgery [12] with the improvement of endothelial function and hemodynamics [13]. We therefore sought to evaluate whether the use of levosimendan could improve weaning of VA-ECMO support in a large cohort of patients undergoing refractory cardiogenic shock.

## Methods

### Study design and patient population

We conducted a retrospective observational study between January 2012 and December 2018 at Louis Pradel University Hospital (Hospices Civils de Lyon, France). The study protocol was approved by our Institutional Review Board (N°20-54; Chair: Prof. JF Guerin) and registered with ClinicalTrial.gov (NCT04323709). Given the retrospective and non-interventional design of the study, the need for written informed consent was waived. All consecutive adult patients admitted to the cardiothoracic intensive care unit (ICU) who underwent VA-ECMO for refractory cardiogenic shock were eligible for the study. Exclusion criteria were age < 18 years, VA-ECMO duration < 48 h, VA-ECMO for refractory cardiac arrest, right heart or veno-venous ECMO, and VA-ECMO for circulatory failure following lung transplantation.

### Data collection

The following data were collected at the admission: age, gender, body mass index, Simplified Acute Physiology Score (SAPS-II), Sequential Organ Failure Assessment (SOFA), hypertension, diabetes, hypercholesterolemia, smoking status, history of stroke or congestive heart failure, coronary or peripheral artery disease, renal failure with dialysis, left ventricular ejection fraction (LVEF), tricuspid annular plane systolic excursion (TAPSE), mean arterial pressure, heart rate, central venous pressure, ScvO_2_, presence of an intra-aortic balloon pump, and biochemical parameters. During the hospitalization, the following variables were collected: the reason for initiation of VA-ECMO and VA-ECMO characteristics (duration, type, flow (L/min), RPM, FiO_2_), length of stay in ICU, catecholamines and inotrope maximal doses and durations of administration, and patients receiving heart transplantation or left ventricular assist device (LVAD). In patients receiving levosimendan, the timing of administration regarding VA-ECMO initiation was also collected.

### Patients’ management

During the study period, all patients were managed according to international guidelines for cardiogenic shock [14]. Timings of administration of levosimendan (Zimino®, Orion Pharma, Issy-les-Moulineaux, France) and catecholamines were at the entire discretion of the physicians. The administration of levosimendan was started at a dose of 0.1 μg/kg/min for 1 h, followed by a continuous infusion of 0.1 to 0.2 μg/kg/min for 24 h. VA-ECMO flow rate was initially set at the theoretical cardiac output owing to the body surface area of the patient (2.2 L/min/m^2^). Inotropic support was usually provided in order to maintain both a left ventricular ejection and an aortic valve opening. Anticoagulation with unfractionated heparin was used to maintain anti-Xa factor activity between 0.30 and 0.35 IU/ml during mechanical support. Serial transesophageal echocardiography was performed after a progressive reduction of VA-ECMO flow to a minimum of 1.0–1.5 L/min to assess myocardial recovery. When the weaning trial was hemodynamically well tolerated without the need for increasing inotropic or vasoactive support and echocardiographic criteria were fulfilled (LVEF > 20–25%, time-velocity integral > 10 cm, lateral mitral annulus peak systolic velocity > 6 cm/s, satisfactory right ventricular systolic function without dilatation [15]), the weaning procedure was performed.

### Study endpoints

The primary endpoint was VA-ECMO weaning failure defined as death occurring during VA-ECMO support or within 24 h after VA-ECMO removal [12, 16]. Secondary endpoints were mortality at day 28 and at 6 months after VA-ECMO implantation. Based on the available literature [17–24] and on the analysis of patient outcomes in our institutional database [25, 26], we classified indications for VA-ECMO into three categories (high, intermediate, or low) according to the potential for myocardial recovery.

### Statistical analysis

Continuous variables were summarized as mean ± standard deviation and compared using Student’s *t* test or Mann–Whitney *U* test depending on their normality. Categorical variables were summarized as counts and percentages and compared using Pearson’s chi-squared test or Fisher’s exact test, as appropriate. Survival at 28 days was reported with Kaplan–Meier curves and compared between the two groups with the log-rank test. We conducted a multivariable logistic regression with propensity score matching [27, 28], which was defined as the probability of exposure to levosimendan. We selected only the covariates most likely to introduce a confounding bias based on clinical expertise and inputs from the literature [20, 29–31]: potential for myocardial recovery, age, gender, SAPS-II, SOFA, LVEF, duration of VA-ECMO, and lactate level. Next, we performed matching with replacement between patients from the levosimendan group and those from the control group in a 1:10 ratio. Finally, we undertook multivariate weighted logistic regression with weaning failure as an outcome variable and the treatment group and the matched variables as explanatory variables. Results were reported as odds ratios (ORs) together with 95% confidence intervals (CIs) assuming a 5% level of statistical significance. All analyses were conducted in STATA 16.0 (Stata Corp, College Station, Texas 77845 USA).

## Results

### Population

**Fig. 1:**
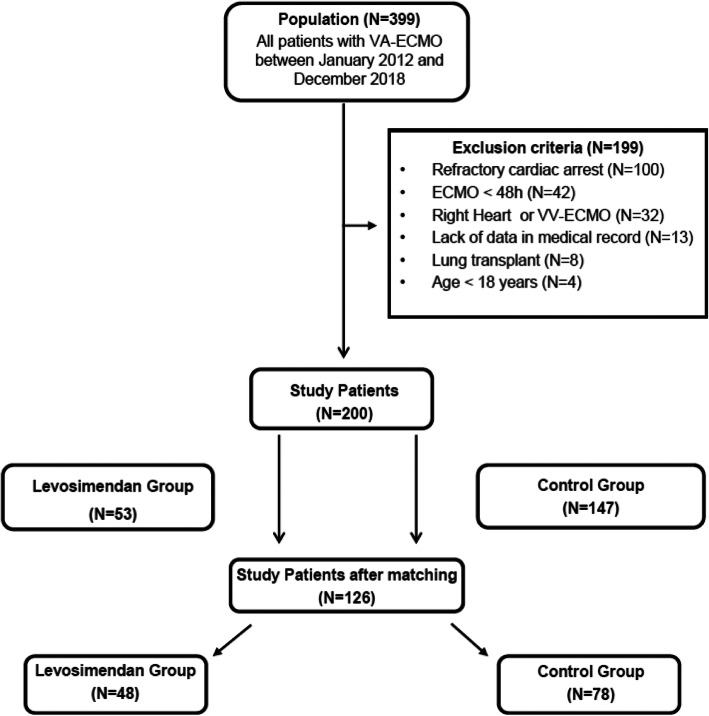
The study flow chart

**Fig. 2:**
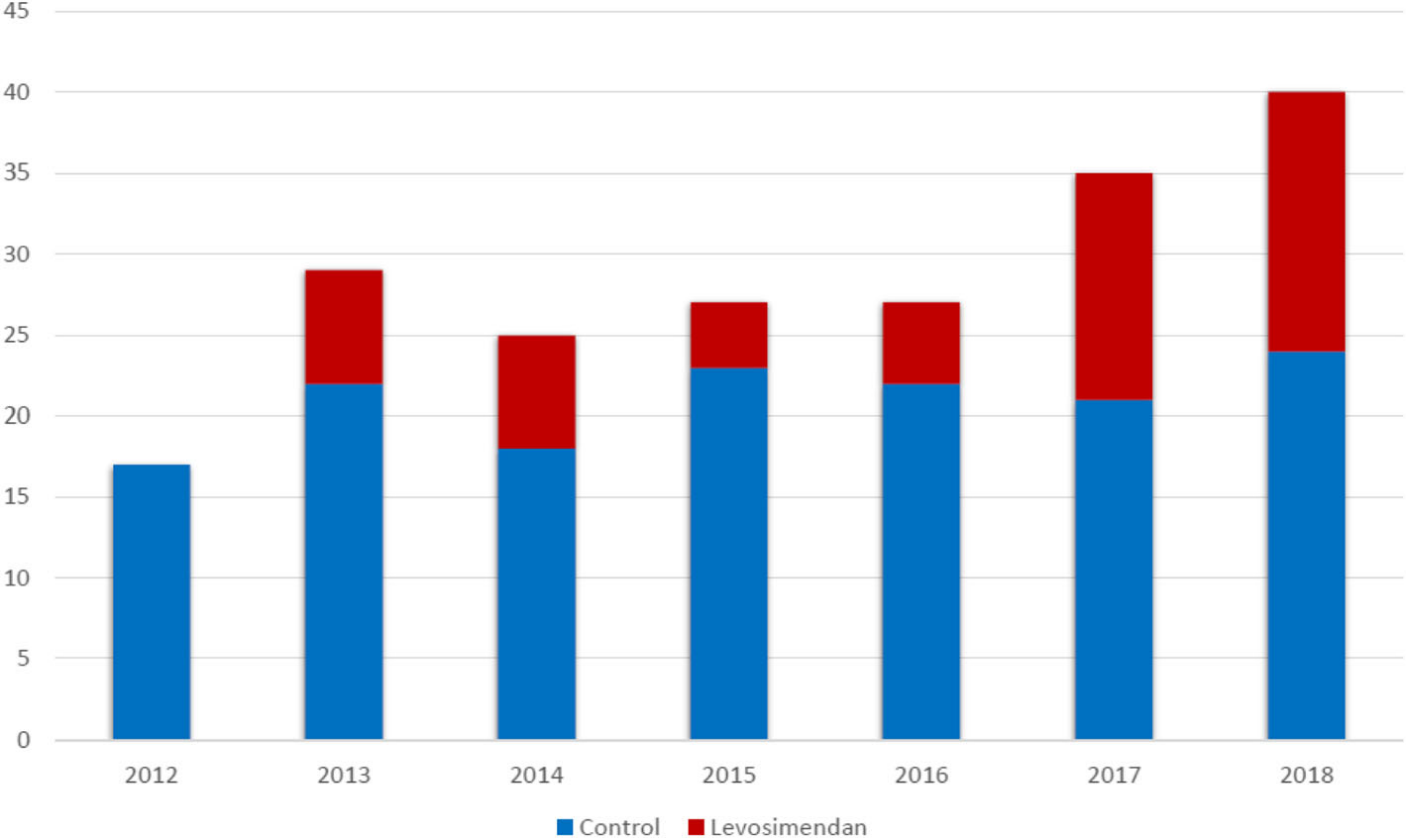
Proportion of patients receiving levosimendan over the study period (2012–2018)

The flow chart of the study is depicted in Fig. 1. Of 399 patients admitted to the ICU who received VA-ECMO, 199 patients were excluded, leaving a total of 200 patients who met eligibility criteria: 53 in the group levosimendan and 147 in the control group. The use of levosimendan in that specific indication started in 2013 and climbed up over time, reaching 40% of VA-ECMO patients in 2018 (Fig. 2).

### Baseline characteristics and outcomes in the unmatched cohort

**Fig. 3:**
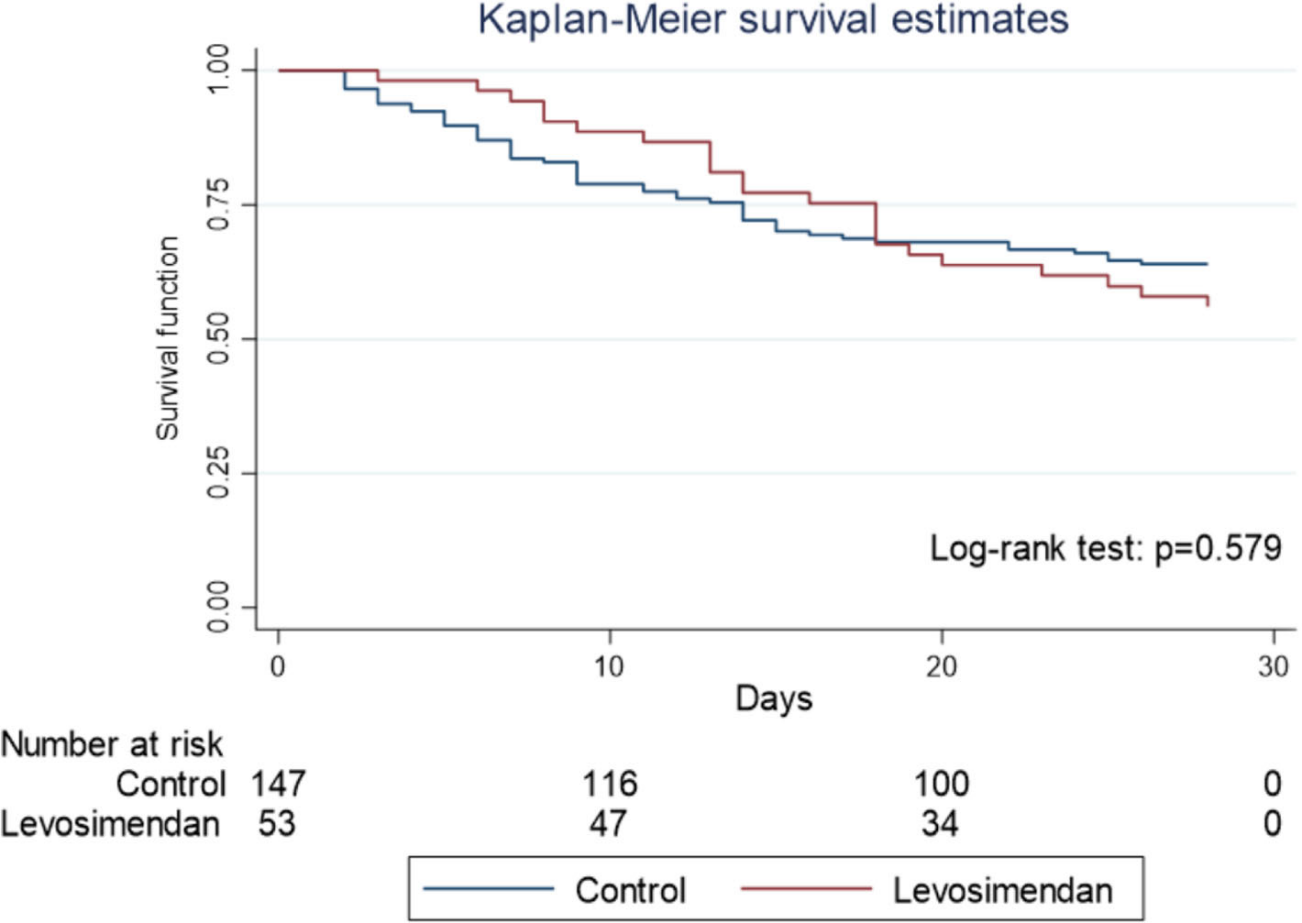
Kaplan-Meier survival curves in the unmatched cohort of patients (*N* = 200)

No significant difference in baseline characteristics was found between groups except for VA-ECMO duration which was longer in the levosimendan group (10.6 ± 4.8 vs. 6.5 ± 4.7 days, *p* < 0.001) (Table 1). Indications for VA-ECMO were mainly represented by post-cardiotomy low cardiac output syndrome (29.5%), acute myocardial infarction (22.5%), and graft dysfunction (16.5%). Mean LVEF at admission was 19.6 ± 11.3%, and 30.8% of patients had a significant right ventricular failure. Peripheral VA-ECMO cannulation was performed in 87.5% of cases and 27% of patients had IABP associated with VA-ECMO. Fifty-three (26.5%) patients received levosimendan, the administration starting at 6.6 ± 5.4 days after implantation. Rates of weaning failure were 28.3% and 29.9% in the levosimendan and control groups, respectively (OR 0.92; 95% CI 0.46–1.85). The mortality rate at 28 days was 44.2% in the levosimendan group and 37.5% in the control group (OR 0.69; 95% CI 0.39–2.51) (Fig. 3). Heart transplantation was more frequent in the levosimendan group (13.7% vs. 4.0% respectively, *p* = 0.017), as was LVAD implantation (11.3% vs. 2.7% respectively, *p* = 0.014). SOFA score and LVEF at admission were the only covariates associated with weaning failure after multivariate analysis (see Appendix).

**Table 1:** Patients demographic and clinical characteristics

	All (*n* = 200)	Levosimendan (*n* = 53)	Control (*n* = 147)	*p* value
Clinical characteristics at ICU admission				
Age (years)	53 ± 13.5	53.9 ± 14.3	52.6 ± 13.3	0.575
Male	129 (64.5)	33 (62.3)	96 (65.3)	0.692
BMI (kg/m^2^)	25.3 ± 5.4	25.3 ± 5.6	25.3 ± 5.3	0.975
SAPS-II	52.2 ± 14.3	53.5 ± 10.8	51.7 ± 15.4	0.349
SOFA	11.7 ± 2.1	11.5 ± 1.5	11.8 ± 2.2	0.459
Comorbidities				
Hypertension	62 (31)	18 (34)	44 (29.9)	0.587
Diabetes	36 (18)	11 (20.8)	25 (17)	0.430
History of congestive heart failure	101 (51)	26 (50)	75 (51.4)	0.865
Coronary artery disease	88 (44)	27 (50.9)	61 (41.5)	0.235
Peripheral artery disease	11 (5.5)	4 (7.5)	7 (4.8)	0.446
History of stroke	14 (7)	5 (9.4)	9 (6.1)	0.418
Smoking	71 (35.5)	18 (34)	53 (36.1)	0.785
Dyslipidemia	49 (24.5)	14 (26.4)	35 (23.8)	0.705
Renal failure with dialysis	15 (7.5)	3 (5.6)	12 (8.1)	0.553
Indication for VA-ECMO				0.068
Post-cardiotomy	59 (29.5)	18 (34)	41 (27.9)	
Acute myocardial infarction	45 (22.5)	17 (32.1)	28 (19)	
Graft dysfunction	33 (16.5)	3 (5.7)	30 (20.4)	
Dilated cardiomyopathy	15 (7.5)	5 (9.4)	10 (6.8)	
Intoxication	14 (7)	0 (0)	14 (9)	
Fulminant myocarditis	13 (6.5)	3 (5.7)	10 (6.8)	
Pulmonary embolism	6 (3)	2 (3.8)	4 (2.7)	
Septic cardiomyopathy	6 (3)	2 (3.8)	4 (2.7)	
Others	9 (4.5)	3 (5.6)	6 (4)	
Potential for myocardial recovery				0.264
High	39 (19.5)	7 (13.2)	32 (21.8)	
Intermediate	86 (43)	22 (41.5)	64 (43.5)	
Low	75 (37.5)	24 (45.3)	51 (34.7)	
Hemodynamic parameters at admission	19.6 ± 11.3	18 ± 11.1	20.2 ± 11.4	0.241
LVEF (%)	45 (30.8)	15 (34.8)	30 (29.1)	0.489
TAPSE < 12 (mm)	69 ± 11	70 ± 11	69 ± 11	0.643
MAP (mmHg)	103 ± 24	108 ± 21	102 ± 25	0.145
HR (beats/min)	10.6 ± 5	10.8 ± 5.5	10.6 ± 4.9	0.798
CVP (mmHg) ScvO_2_ (%)	62 ± 11	60 ± 12	63 ± 11	0.065
VA-ECMO characteristics				
VA-ECMO duration (days)	7.6 ± 5	10.6 ± 4.8	6.5 ± 4.7	**< 0.001**
Flow rate (L/min)	3.5 ± 0.8	3.4 ± 0.8	3.5 ± 0.8	0.835
Rotation (round/min)	4360 ± 1711	4480 ± 1724	4312 ± 1710	0.547
FiO_2_ (%)	59 ± 12	58 ± 12	59 ± 13	0.977
Peripheral VA-ECMO canulation	175 (87.5)	48 (90.6)	127 (86.4)	0.496
IABP associated to VA-ECMO	54 (27)	16 (30.1)	38 (25.8)	0.542
Biological parameters				
Hemoglobin level (g/dL)	113 ± 25	114 ± 26	113 ± 24	0.717
International normalized ratio	1.6 ± 0.6	1.5 ± 0.5	1.6 ± 0.6	0.338
Arterial blood pH	7.26 ± ± 0.1	7.27 ± 0.1	7.26 ± 0.1	0.652
Lactate level (mmol/L)	7.2 ± 5.1	6.4 ± 4.7	7.5 ± 5.3	0.178
Creatinine level (μmol/L)	152 ± 78	150 ± 77	153 ± 79	0.843
Total bilirubin level (μmol/L)	23 ± 17	22 ± 17	24 ± 17	0.457
ASAT (U/L)	763 ± 1819	717 ± ± 1454	781 ± 1945	0.828
ALAT (U/L)	390 ± 907	295 ± 610	426 ± 998	0.372
Catecholamines during ICU stay				
Norepinephrine max dose (μg/kg/min)	1.49 ± 1.05	1.56 ± 1.07	1.47 ± 1.04	0.586
Norepinephrine duration (days)	10.9 ± 8.7	12.8 ± 7.2	10.2 ± 9.2	0.068
Dobutamine max dose (μg/kg/min)	9.7 ± 4.6	10.4 ± 10.2	9.5 ± 4.3	0.309
Dobutamine duration (days)	9.1 ± 7.9	10.3 ± 10.2	8.6 ± 6.6	0.203

*ICU* intensive care unit, *BMI* body mass index, *SAPS-II* simplified acute physiology score, *SOFA* sequential organ failure assessment, *LVEF* left ventricular ejection fraction, *TAPSE* tricuspid annular plane systolic excursion, *MAP* mean arterial pressure, *HR* heart rate, *CVP* central venous pressure, *ScvO*_*2*_ central venous oxygen saturation, *FiO*_*2*_ fractional inspired oxygen, *IABP* intra-aortic balloon pump, *ASAT* aspartate aminotransferase, *ALAT* alanine aminotransferase

### Baseline characteristics and outcomes after propensity score analysis

After matching of 48 patients in the levosimendan group to 78 in the control group (Fig. 1), the balance of covariates was improved with no statistical difference on any of the covariates, including VA-ECMO duration (Table 2). Rates of weaning failure were 29.1% and 35.4% in the levosimendan and control groups, respectively (OR 0.69; 95% CI 0.25–1.88). No significant difference was found between both groups for all secondary outcomes. Rates of death at 28 days were 41.0% and 41.6% in the levosimendan and the control groups, respectively (OR 1.08; 95% CI 0.42–2.81). Rates of death at 6 months were 50.0% and 54.3% in the levosimendan and control groups, respectively (OR 0.79; 95% CI 0.30–2.07).

**Table 2:** Balance of covariates before and after matching

	Unmatched*			Matched		
	Levosimendan (*n* = 48)	Control (*n* = 128)	*p*	Levosimendan (*n* = 48)	Control (*n* = 78)	*p*
**Variable (mean)**						
Age (years)	53.9	52.6	0.575	54.3	54.7	0.866
Male (%)	62	65	0.692	0.62	0.65	0.785
Potential for recovery	2.32	2.12	0.104	2.31	2.35	0.747
SAPS-II	53.5	51.7	0.424	52.7	52.1	0.824
SOFA	11.5	11.8	0.530	11.3	11.5	0.687
LVEF (%)	18	20.2	0.241	18	17	0.690
VA-ECMO duration (days)	10.6	6.5	**0.000**	10.8	10.2	**0.478**
Serum lactate level (mmol/L)	6.4	7.5	0.178	6.3	6.1	0.816

Myocardial recovery potential: *High 1* intermediate 2, *Low 3 SAPS-II* simplified acute physiology score, *SOFA* sequential organ failure assessment, *LVEF* left ventricular ejection fraction. Data are expressed as mean. The *p* value refers to a comparison between the levosimendan group and the control group. *Compared to the entire cohort (*n* = 200), the unmatched population had 176 patients since there were 24 patients with missing data on some of the variables used in the analysis

## Discussion

The main finding of the present study is that levosimendan did not significantly improve the rate of VA-ECMO weaning success in ICU patients with refractory cardiogenic shock. Moreover, no benefit of levosimendan was found on mortality at 28 days and 6 months after admission.

The theoretical specific features of levosimendan are of interest in such a clinical setting: (1) inotropic effect with respect to myocardial oxygen balance; (2) lack of pro-arrhythmic effect or interaction with beta-blockers; (3) systemic, pulmonary, and coronary vasodilation; and (4) cardioprotective effect against ischemia/reperfusion injury as well as anti-inflammatory properties [10]. Moreover, its long-lasting action (up to 8–9 days) due to circulating active metabolites could be particularly useful by providing a continuous support in the critical immediate post-VA-ECMO period. A first pilot case-control study including 17 patients undergoing VA-ECMO for the cardiogenic shock of varying etiologies was published in 2013 and found some benefit when levosimendan was used 24 h before the planned weaning [32]. However, the small sample size of that study (6 patients only received levosimendan) did not allow any definite conclusion. More recently, the study conducted by Distelmaier et al. also suggested a beneficial effect of levosimendan on a large population of 240 cardiac surgical patients experiencing postoperative low cardiac output syndrome [12]. Indeed, a strong association was found between levosimendan and both successful VA-ECMO weaning and short- and long-term mortality. Conversely, the study by Jacky et al. conducted in the setting of cardiac surgery compared levosimendan to milrinone without any difference between the two drugs [16]. Recently, Vally et al. reported that exposure to levosimendan might be independently associated with beneficial effects on peripheral VA-ECMO weaning in patients with refractory cardiogenic shock [33]. The survival rate at 30 days was increased in patients receiving levosimendan only in the unmatched analysis [33].

In the current study, we used a propensity score analysis with a choice of variables to include based upon a comparison of baseline characteristics between the two groups, inputs from both literature and clinical expertise. No statistical difference in any of the included covariates was found between the 126 matched patients. One of the covariates included in the propensity score was the potential for myocardial recovery based upon indications for VA-ECMO. Indeed, outcomes of patients undergoing VA-ECMO greatly differ regarding the reason for ECMO implantation [17–26]. Forty-two patients who received ECMO less than 48 h were excluded because we considered the probability to receive levosimendan was too low. Patients with refractory cardiac arrest were also excluded because of a high-mortality rate in our institution [26] and the specific pathophysiology of post-cardiac arrest syndrome. In a large observational study, the rate of successful weaning in 4658 patients with cardiogenic shock was reported to be limited to 65.7% [30], a result pretty similar to our findings.

### Limitations

Our study suffers several obvious limitations such as its observational nature and a possible lack of power due to the low number of patients included in the matched analysis. Although we have used propensity score matching to reduce selection bias, there is still a risk that our two groups may not be comparable due to the presence of confounding variables not accounted for in our model (unknown or unmeasured confounders) [34]. Thus, the results observed here may not be reproducible within the scope of a randomized controlled trial (RCT). More broadly, observational studies and RCT can generate heterogenous or even conflicting results [35]. Given this limitation, our findings should be viewed cautiously and call for future clinical research using a more robust design. Moreover, in the levosimendan group, 34.8% of patients presented a TAPSE < 12 mm at ICU admission compared to 29.1% in the control group. Although not significant, this result may suggest a higher proportion of patients with right ventricular dysfunction in the levosimendan group and may have contributed to limit the effect of levosimendan on VA-ECMO weaning success in patients presenting bi-ventricular failure [36]. Also, we observed an increasing use of levosimendan over the most recent period with the risk that the variation in team performance over time may have led to minimize the drug effect on VA-ECMO weaning. Another main limitation is that administration of levosimendan occurred late after VA-ECMO implantation. This timing could be too late when compared with other studies [12, 33]. We postulate that physicians started levosimendan in a second step, only in patients demonstrating a weak probability of ECMO weaning success. Many arguments support that hypothesis. First, the duration of VA-ECMO was significantly longer in the levosimendan group in the unmatched analysis. Second, even if not statistically significant, a greater proportion of patients with a high potential for myocardial recovery did not receive levosimendan (control group). Third, more patients in the levosimendan group had heart transplantation or LVAD. By contrast, levosimendan was administered only 3 days after VA-ECMO start in the study reported by Vally et al. [33], a shorter delay that may have contributed to their positive results. Finally, as a tertiary care university hospital with high volumes for transplantation and LVAD, we are more exposed to treat patients with severe refractory cardiogenic shock and supported with VA-ECMO for many days prior to admission in our institution.

## Conclusion

In conclusion, the current study found no benefit to levosimendan in order to reduce VA-ECMO weaning failure in a population of patients with surgical and medical refractory cardiogenic shocks. Facing the discordance between the most recent data, there is an urgent need for a large randomized clinical trial which could bring more reliable information regarding the interest of levosimendan in that clinical setting, if any.

## Supplementary information

**Additional file 1.**

## Data Availability

The data used and/or analyzed in the present study are available from the corresponding author on reasonable request.

## Abbreviations

Cis: Confidence intervals
ICU: Intensive care unit
LVAD: Left ventricular assist device
LVEF: Left ventricular ejection fraction
ORs: Odds ratios
RCT: Randomized controlled trial
RPM: Rotation per minute
SAPS-II: Simplified acute physiology score
ScvO_2_: Central venous oxygen saturation
SOFA: Sequential organ failure assessment
TAPSE: Tricuspid annular plane systolic excursion
VA-ECMO: Veno-arterial extracorporeal membrane oxygenation

## References

[1] Ventetuolo CE, Muratore CS (2014). Extracorporeal life support in critically ill adults. Am J Respir Crit Care Med.

[2] Extracorporeal Life Support Organization. ELSO guidelines for adult cardiac failure v1.3. Michigan, USA, 2015. http://www.elso.org/Resources/Guidelines. aspx. Accessed 1 July 2015.

[3] Allou N, Lo Pinto H, Persichini R, Bouchet B, Braunberger E, Lugagne N (2019). Cannula-related infection in patients supported by peripheral ECMO: clinical and microbiological characteristics. ASAIO J.

[4] Aubron C, DePuydt J, Belon F, Bailey M, Schmidt M, Sheldrake J (2016). Predictive factors of bleeding events in adults undergoing extracorporeal membrane oxygenation. Ann Intensive Care.

[5] Trudzinski FC, Minko P, Rapp D, Fähndrich S, Haake H, Haab M (2016). Runtime and aPTT predict venous thrombosis and thromboembolism in patients on extracorporeal membrane oxygenation: a retrospective analysis. Ann Intensive Care.

[6] Levy B, Bastien O, Karim B, Cariou A, Chouihed T, Combes A, Mebazaa A, Megarbane B, Plaisance P, Ouattara A, Spaulding C, Teboul JL, Vanhuyse F, Boulain T, Kuteifan K (2015). Experts’ recommendations for the management of adult patients with cardiogenic shock. Ann Intensive Care.

[7] Ellender T, Skinner J (2008). The use of vasopressors and inotropes in the emergency medical treatment of shock. Emerg Med Clin N Am.

[8] Haikala H, Kaivola J, Nissinen E, Wall P, Levijoki J, Linden IB (1995). Cardiac troponin C as a target protein for a novel calcium sensitizing drug, levosimendan. J Mol Cell Cardiol.

[9] Erdei N, Papp Z, Pollesello P (2006). The levosimendan metabolite OR- 1896 elicits vasodilation by activating the K (ATP) and BK (Ca) channels in rat isolated arterioles. Br J Pharmacol.

[10] Pathak A, Lebrin M, Vaccaro A, Senard JM, Despas F (2013). Pharmacology of levosimendan: inotropic, vasodilatory and cardioprotective effects. J Clin Pharm Ther.

[11] Cholley B, Levy B, Fellahi JL, Longrois D, Amour J, Ouattara A, Mebazaa A (2019). Levosimendan in the light of the results of the recent randomized controlled trials: an expert opinion paper. Crit Care.

[12] Distelmaier K, Roth C, Schrutka L (2016). Beneficial effects of levosimendan on survival in patients undergoing extracorporeal membrane oxygenation after cardiovascular surgery. Br J Anaesth.

[13] Sangalli F, Avalli L, Laratta M (2016). Effects of levosimendan on endothelial function and hemodynamics during weaning from venoarterial extracorporeal life support. J Cardiothorac Vasc Anesth.

[14] van Diepen S, Katz JN, Albert NM, Henry TD, Jacobs AK, Kapur NK, Kilic A, Menon V, Ohman EM, Sweitzer NK, Thiele H, Washam JB, Cohen MG (2017). Contemporary management of cardiogenic shock: a scientific statement from the American Heart Association. Circulation..

[15] Aissaoui N, Luyt CE, Leprince P, Trouillet JL, Léger P, Pavie A (2011). Predictors of successful extracorporeal membrane oxygenation (ECMO) weaning after assistance for refractory cardiogenic shock. Intensive Care Med.

[16] Jacky A, Rudiger A, Krüger B (2018). Comparison of levosimendan and milrinone for ECLS weaning in patients after cardiac surgery—a retrospective before and after study. J Cardiothorac Vasc Anesth.

[17] Cheng R, Hachamovitch R, Kittleson M, Patel J, Arabia F, Moriguchi J, Esmailian F, Azarbal B (2014). Clinical outcomes in fulminant myocarditis requiring extracorporeal membrane oxygenation: a weighted meta-analysis of 170 patients. J Card Fail.

[18] Lorusso R, Centofanti P, Gelsomino S (2016). Venoarterial extracorporeal membrane oxygenation for acute fulminant myocarditis in adult patients: a 5-year multi-institutional experience. Ann Thorac Surg.

[19] Masson R, Colas V, Parienti JJ, Lehoux P, Massetti M, Charbonneau P, Saulnier F, Daubin C (2012). A comparison of survival with and without extracorporeal life support treatment for severe poisoning due to drug intoxication. Resuscitation..

[20] Muller G, Flecher E, Lebreton G, Luyt CE, Trouillet JL, Bréchot N, Schmidt M, Mastroianni C, Chastre J, Leprince P, Anselmi A, Combes A (2016). The ENCOURAGE mortality risk score and analysis of long-term outcomes after VA-ECMO for acute myocardial infarction with cardiogenic shock. Intensive Care Med.

[21] Pabst D, Foy AJ, Peterson B, Soleimani B, Brehm CE (2018). Predicting survival in patients treated with extracorporeal membrane oxygenation after myocardial infarction. Crit Care Med.

[22] Biancari F, Perrotti A, Dalén M, Guerrieri M, Fiore A, Reichart D, Dell'Aquila AM, Gatti G, Ala-Kokko T (2018). Meta-analysis of the outcome after postcardiotomy venoarterial extracorporeal membrane oxygenation in adult patients. J Cardiothorac Vasc Anesth.

[23] Dangers L, Bréchot N, Schmidt M, Lebreton G, Hékimian G, Nieszkowska A, Besset S, Trouillet JL, Chastre J, Leprince P, Combes A, Luyt CE (2017). Extracorporeal membrane oxygenation for acute decompensated heart failure. Crit Care Med.

[24] Ouweneel DM, Schotborgh JV (2016). Extracorporeal life support during cardiac arrest and cardiogenic shock: a systematic review and meta-analysis. Intensive Care Med.

[25] Pozzi M, Bottin C, Armoiry X, Sebbag L, Boissonnat P, Hugon-Vallet E, Koffel C, Flamens C, Paulus S, Fellahi JL, Obadia JF (2018). Extracorporeal life support for primary graft dysfunction after heart transplantation. Interact Cardiovasc Thorac Surg.

[26] Pozzi M, Armoiry X, Achana F, Koffel C, Pavlakovic I, Lavigne F, Fellahi JL, Obadia JF (2019). Extracorporeal life support for refractory cardiac arrest: a 10-year comparative analysis. Ann Thorac Surg.

[27] Sascha O. Becker and Andrea Ichino. Estimation of average treatment effects based on propensity scores February 2002 Stata Journal 2(4):358–377.

[28] Leuven, E., and B. Sianesi. 2003. “PSMATCH2: Stata module to perform full Mahalanobis and propensity score matching, common support graphing, and covariate imbalance testing, version 4.0.6”).

[29] Lee HS, Kim HS, Lee SH, Lee SA, Hwang JJ, Park JB, Kim YH, Moon HJ, Lee WS (2019). Clinical implications of the initial SAPS II in veno-arterial extracorporeal oxygenation. J Thorac Dis.

[30] Aso S, Matsui H, Fushimi K, Yasunaga H (2016). In-hospital mortality and successful weaning from venoarterial extracorporeal membrane oxygenation: analysis of 5263 patients using a national inpatient database in Japan. Crit Care.

[31] Smith M, Vukomanovic A, Brodie D, Thiagarajan R, Rycus P, Buscher H (2017). Duration of veno-arterial extracorporeal life support (VA ECMO) and outcome: an analysis of the Extracorporeal Life Support Organization (ELSO) registry. Crit Care.

[32] Affronti A, di Bella I, Carino D (2013). Levosimendan may improve weaning outcomes in venoarterial ECMO patients. ASAIO J.

[33] Vally S, Ferdynus C, Persichini R, Bouchet B, Braunberger E, Lo Pinto H, Martinet O, Vandroux D, Aujoulat T, Allyn J, Allou N (2019). Impact of levosimendan on weaning from peripheral venoarterial extracorporeal membrane oxygenation in intensive care unit. Ann Intensive Care.

[34] Nuttall GA, Houle TT (2008). Liars, damn liars, and propensity scores. Anesthesiology..

[35] Armoiry X, Obadia JF (2018). Comparison of transcatheter versus surgical aortic valve implantation in high-risk patients: a nationwide study in France. J Thorac Cardiovasc Surg.

[36] Pappalardo F, Pieri M (2015). Timing and strategy for weaning from venoarterial ECMO are complex issues. J Cardiothorac Vasc Anesth.

