# Correction to: Can levosimendan reduce ECMO weaning failure in cardiogenic shock?: a cohort study with propensity score analysis

**DOI:** 10.1186/s13054-020-03213-w

**Published:** 2020-08-05

**Authors:** Enrique Guilherme, Matthias Jacquet-Lagrèze, Matteo Pozzi, Felix Achana, Xavier Armoiry, Jean-Luc Fellahi

**Affiliations:** 1 Hospices Civils de Lyon, Hôpital Louis Pradel, Service d’Anesthésie-Réanimation, Lyon, France; 2 INSERM U1060, Laboratoire CarMeN, IHU OPeRa, Lyon, France; 3 Hospices Civils de Lyon, Hôpital Louis Pradel, Service de Chirurgie Cardiaque, Lyon, France; 4 Nuffield Department of Primary care, Oxford University, Oxford, UK; 5 Lyon School of Pharmacy (ISPB), Public Health department/UMR CNRS 5510 MATEIS, I2B Team, Lyon, France; 6 Division of Health Sciences, Warwick Medical School, Warwick university, Coventry, UK

**Correction to: Crit Care 24, 442 (2020)**

**https://doi.org/10.1186/s13054-020-03122-y**

Following publication of the original article [1], the authors reported errors in Table 1 and Table 2.

The revised Table 1 and Table 2 are indicated hereafter.

**Table 1:** Patients demographic and clinical characteristics

	All (*n* = 200)	Levosimendan (*n* = 53)	Control (*n* = 147)	*p* value
Clinical characteristics at ICU admission				
Age (years)	53 ± 13.5	53.9 ± 14.3	52.6 ± 13.3	0.575
Male	129 (64.5)	33 (62.3)	96 (65.3)	0.692
BMI (kg/m^2^)	25.3 ± 5.4	25.3 ± 5.6	25.3 ± 5.3	0.975
SAPS-II	52.2 ± 14.3	53.5 ± 10.8	51.7 ± 15.4	0.349
SOFA	11.7 ± 2.1	11.5 ± 1.5	11.8 ± 2.2	0.459
Comorbidities				
Hypertension	62 (31)	18 (34)	44 (29.9)	0.587
Diabetes	36 (18)	11 (20.8)	25 (17)	0.430
History of congestive heart failure	101 (51)	26 (50)	75 (51.4)	0.865
Coronary artery disease	88 (44)	27 (50.9)	61 (41.5)	0.235
Peripheral artery disease	11 (5.5)	4 (7.5)	7 (4.8)	0.446
History of stroke	14 (7)	5 (9.4)	9 (6.1)	0.418
Smoking	71 (35.5)	18 (34)	53 (36.1)	0.785
Dyslipidemia	49 (24.5)	14 (26.4)	35 (23.8)	0.705
Renal failure with dialysis	15 (7.5)	3 (5.6)	12 (8.1)	0.553
Indication for VA-ECMO				0.068
Post-cardiotomy	59 (29.5)	18 (34)	41 (27.9)	
Acute myocardial infarction	45 (22.5)	17 (32.1)	28 (19)	
Graft dysfunction	33 (16.5)	3 (5.7)	30 (20.4)	
Dilated cardiomyopathy	15 (7.5)	5 (9.4)	10 (6.8)	
Intoxication	14 (7)	0 (0)	14 (9)	
Fulminant myocarditis	13 (6.5)	3 (5.7)	10 (6.8)	
Pulmonary embolism	6 (3)	2 (3.8)	4 (2.7)	
Septic cardiomyopathy	6 (3)	2 (3.8)	4 (2.7)	
Others	9 (4.5)	3 (5.6)	6 (4)	
Potential for myocardial recovery				0.264
High	39 (19.5)	7 (13.2)	32 (21.8)	
Intermediate	86 (43)	22 (41.5)	64 (43.5)	
Low	75 (37.5)	24 (45.3)	51 (34.7)	
Hemodynamic parameters at admission				
LVEF (%)	19.6 ± 11.3	18 ± 11.1	20.2 ± 11.4	0.241
TAPSE < 12 (mm)	45 (30.8)	15 (34.8)	30 (29.1)	0.489
MAP (mmHg)	69 ± 11	70 ± 11	69 ± 11	0.643
HR (beats/min)	103 ± 24	108 ± 21	102 ± 25	0.145
CVP (mmHg)	10.6 ± 5	10.8 ± 5.5	10.6 ± 4.9	0.798
ScvO_2_ (%)	62 ± 11	60 ± 12	63 ± 11	0.065
VA-ECMO characteristics				
VA-ECMO duration (days)	7.6 ± 5	10.6 ± 4.8	6.5 ± 4.7	**< 0.001**
Flow rate (L/min)	3.5 ± 0.8	3.4 ± 0.8	3.5 ± 0.8	0.835
Rotation (round/min)	4360 ± 1711	4480 ± 1724	4312 ± 1710	0.547
FiO_2_ (%)	59 ± 12	58 ± 12	59 ± 13	0.977
Peripheral VA-ECMO canulation	175 (87.5)	48 (90.6)	127 (86.4)	0.496
IABP associated to VA-ECMO	54 (27)	16 (30.1)	38 (25.8)	0.542
Biological parameters				
Hemoglobin level (g/dL)	113 ± 25	114 ± 26	113 ± 24	0.717
International normalized ratio	1.6 ± 0.6	1.5 ± 0.5	1.6 ± 0.6	0.338
Arterial blood pH	7.26 ± ± 0.1	7.27 ± 0.1	7.26 ± 0.1	0.652
Lactate level (mmol/L)	7.2 ± 5.1	6.4 ± 4.7	7.5 ± 5.3	0.178
Creatinine level (μmol/L)	152 ± 78	150 ± 77	153 ± 79	0.843
Total bilirubin level (μmol/L)	23 ± 17	22 ± 17	24 ± 17	0.457
ASAT (U/L)	763 ± 1819	717 ± ± 1454	781 ± 1945	0.828
ALAT (U/L)	390 ± 907	295 ± 610	426 ± 998	0.372
Catecholamines during ICU stay				
Norepinephrine max dose (μg/kg/min)	1.49 ± 1.05	1.56 ± 1.07	1.47 ± 1.04	0.586
Norepinephrine duration (days)	10.9 ± 8.7	12.8 ± 7.2	10.2 ± 9.2	0.068
Dobutamine max dose (μg/kg/min)	9.7 ± 4.6	10.4 ± 10.2	9.5 ± 4.3	0.309
Dobutamine duration (days)	9.1 ± 7.9	10.3 ± 10.2	8.6 ± 6.6	0.203

*ICU* intensive care unit, *BMI* body mass index, *SAPS-II* simplified acute physiology score, *SOFA* sequential organ failure assessment, *LVEF* left ventricular ejection fraction, *TAPSE* tricuspid annular plane systolic excursion, *MAP* mean arterial pressure, *HR* heart rate, *CVP* central venous pressure, *ScvO*_*2*_ central venous oxygen saturation, *FiO*_*2*_ fractional inspired oxygen, *IABP* intra-aortic balloon pump, *ASAT* aspartate aminotransferase, *ALAT* alanine aminotransferase

**Table 2:** Balance of covariates before and after matching

	Unmatched*			Matched		
	Levosimendan (*n* = 48)	Control (*n* = 128)	*p*	Levosimendan (*n* = 48)	Control (*n* = 78)	*p*
**Variable (mean)**						
Age (years)	53.9	52.6	0.575	54.3	54.7	0.866
Male (%)	62	65	0.692	0.62	0.65	0.785
Potential for recovery	2.32	2.12	0.104	2.31	2.35	0.747
SAPS-II	53.5	51.7	0.424	52.7	52.1	0.824
SOFA	11.5	11.8	0.530	11.3	11.5	0.687
LVEF (%)	18	20.2	0.241	18	17	0.690
VA-ECMO duration (days)	10.6	6.5	**< 0.001**	10.8	10.2	**0.478**
Serum lactate level (mmol/L)	6.4	7.5	0.178	6.3	6.1	0.816

Myocardial recovery potential: *High 1* intermediate 2, *Low 3 SAPS-II* simplified acute physiology score, *SOFA* sequential organ failure assessment, *LVEF* left ventricular ejection fraction. Data are expressed as mean. The *p* value refers to a comparison between the levosimendan group and the control group. *Compared to the entire cohort (*n* = 200), the unmatched population had 176 patients since there were 24 patients with missing data on some of the variables used in the analysis

The original article [1] has been updated.
