# Supplementary material for: Can levosimendan reduce ECMO weaning failure in cardiogenic shock?: a cohort study with propensity score analysis
Source: Crit Care. 2020 Jul 16;24:442. doi: 10.1186/s13054-020-03122-y (PMC7367381; doi:10.1186/s13054-020-03122-y)

**Appendix**

**Additional file 1. Multivariate analysis conducted on potential confounding variables with p-value < 0.10 in the univariate analysis**

| **Variable** | **Odds ratio with 95% CI** | **p-value** |
| --- | --- | --- |
| Potential for recovery | OR 1.78; 95% CI: 0.90-3.50 | 0.094 |
| Age | OR 1.00; 95% CI: 0.96-1.03 | 0.852 |
| Male | OR 1.61; 95% CI: 0.67-3.83 | 0.280 |
| BMI | OR 1.06; 95% CI: 0.99-1.14 | 0.075 |
| **SOFA** | **OR 1.23; 95% CI: 1.01-1.49** | **0.038** |
| History of stroke | OR 3.53; 95% CI: 0.94-13.24 | 0.061 |
| Coronary artery disease | OR 2.18; 95% CI: 0.93-5.14 | 0.073 |
| History of congestive heart failure | OR 1.65; 95% CI: 0.73-3.74 | 0.226 |
| **LVEF at admission** | **OR 1.03; 95% CI: 1.00-1.07** | **0.032** |

**Additional file 2. Balance of Propensity Score across Treatment**

**Additional file 3. Balance of covariates before and after matching**


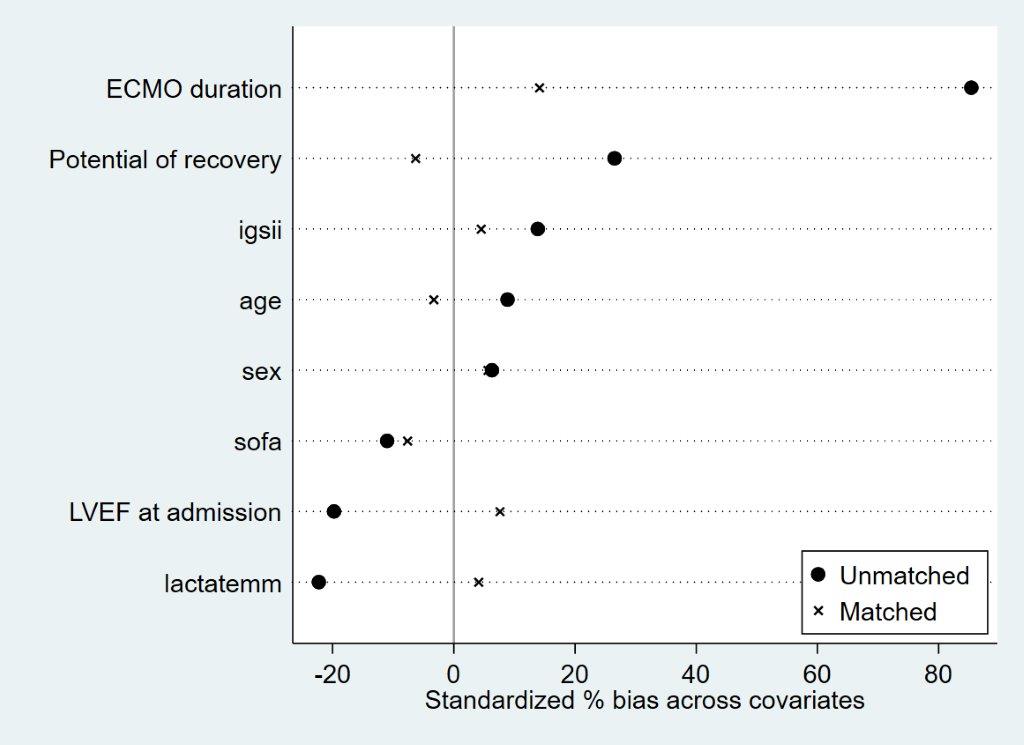

Supplement: Supplementary file 1 — Additional file 1. [file 13054_2020_3122_MOESM1_ESM.docx]
